# Could tumour volume and major and minor axis based on CTA statistical anatomy improve the pre‐operative T‐stage in oesophageal cancer?

**DOI:** 10.1002/cam4.6051

**Published:** 2023-06-12

**Authors:** Runyuan Wang, Xiaoqin Zhang, Wei Wu, Jinfeng Ma, Jincheng Chen, Zhu Zhang, Liqun Liu, Shanshan Xu, Ximei Cao, Yi Wu, Huilin Cui

**Affiliations:** ^1^ Department of Digital Medicine College of Biomedical Engineering and Medical Imaging Army Medical University (Third Military Medical University) Chongqing China; ^2^ Department of Histology and Embryology Shanxi Medical University Taiyuan China; ^3^ Department of Thoracic Surgery, Southwest Hospital Army Medical University (Third Military Medical University) Chongqing China; ^4^ Department of General Surgery, Shanxi Province Cancer Hospital/Shanxi Hospital Affiliated to Cancer Hospital Chinese Academy of Medical Sciences/Cancer Hospital Affiliated to Shanxi Medical University Taiyuan China; ^5^ Department of Obstetrics and Gynecology, Southwest Hospital Army Medical University (Third Military Medical University) Chongqing China

**Keywords:** 3D reconstruction, computed tomography angiography (CTA), oesophageal carcinoma, statistical anatomy, volume

## Abstract

**Objectives:**

To statistically study the 3D shape of oesophageal cancer (EC) and its spatial relationships based on computed tomography angiography (CTA) 3D reconstruction, to determine its relationship with T‐stages, and to create an optimal T‐stage diagnosis protocol based on CTA calculation.

**Methods:**

Pre‐operative CTA images of 155 patients with EC were retrospectively collected and divided into four groups: T1–T4. We used Amira software to segment and 3D reconstruct the EC, oesophagus, aorta, pericardium and peripheral lymph nodes and measured their surface area, volume, major axis, minor axis, longitudinal length, roughness and relationship to the aorta of the EC. One‐way ANOVA, independent sample *t*‐test, ROC, etc., were performed and critical values between different T‐stages were calculated. We also invited two radiologists to evaluate the measurements.

**Results:**

There were no significant differences in EC longitudinal length, roughness score and relationship with the aorta between the different T‐stages of EC. There were significant differences in EC surface area, EC volume and mean major and minor axis among the different T‐stages. The volumes of the T1–T4 tumours were 12,934.36 ± 7739.25, 23,095.27 ± 14,975.67, 37,577.98 ± 36,085.64 and 58,579.25 ± 41,073.96 mm^3^ separately (*p* < 0.05), and the T1–T4 volume cut‐off values were 11,712.00, 19,809.00 and 44,103.50 mm^3^ separately. For comparison with radiologists, the AUC value of our measurements was 0.704, which was higher than the radiologists of AUC = 0.630.

**Conclusions:**

EC volume, major and minor axis can be used as important factors for surgeons in the T‐stage diagnosis of EC, which helps to improve prognosis and treatment decisions after CTA.

## 1 INTRODUCTION

Oesophageal cancer (EC) is one of the most malignant diseases worldwide, with the seventh highest incidence and sixth highest overall mortality rate,^1^ mainly owing to its later diagnosis, rapid progression and poor prognosis.^2^, ^3^ According to statistics, one in every 18 cancer deaths in 2020 will be due to EC,^1^ and the 5‐year survival rate for advanced EC is only 15%–25%,^4^ which seriously endangers human survival and quality of life.^5^, ^6^, ^7^ Clinically, the diagnosis and staging of pre‐operative EC mainly rely on computed tomography angiography (CTA) which can assess the location and extent of oesophageal tumours, and also formulate the optimal treatment plan for patients based on T staging. However, In CTA images, it is difficult for radiologists and surgeons to T‐stage diagnose EC, especially for early stage tumours (T1a, T1b).^9^, ^10^ At present, the T‐staging of EC also relies on Endoscopic Ultrasound (EUS), Positron emission tomography/Computational tomography (PET/CT)^40^ and post‐surgical pathology. EUS is the preferred method for assessing T‐stage of oesophageal cancer with an accuracy between 70% and 90%.^39^ However, EUS also has a high error rate, even more than 50% and a low accuracy at T1 and T2 stages, there examinations are costly, invasive and prone to sampling errors.^14^, ^38^ Therefore, the diagnosis of pre‐operative T‐staging of EC is key to treatment and survival for all patients.^8^

In recent years, 3D reconstruction technology has developed rapidly, which is based on CTA or magnetic resonance imaging (MRI) images, combined with computer technology and algorithms, comprehensively and revealing the location, 3D morphology and spatial relationships including arteries, veins, lymph nodes and normal organs surrounding the tumour.^11^, ^12^, ^13^, ^14^ Cai et al.^13^ used 3D reconstruction technology in the diagnosis and treatment of patients with lower oesophageal tumours, which plays an important role in the pre‐operative assessment of the tumour and its adjacent lymph nodes. Xu et al.,^37^ the results of their study suggest that oesophageal tumour length and diameter determined by radiography are valuable prognostic factors for ESCC patients undergoing definitive (chemo)radiotherapy. Currently, tumour imaging and 3D volumetric measurements are mostly employed to predict T‐stage in colorectal cancer,^15^ nasopharyngeal Carcinoma^16^ and non‐small cell lung cancer,^17^ which helps in accurate diagnosis and prognostic assessment but are less common in predicting T‐stage in EC.

Therefore, we performed a retrospective study involving 155 patients with EC, and built 3D models of EC through their thoracic CTA images and 3D reconstruction technology, and studied statistical anatomical morphology of EC and its adjacent structures, aiming to find some morphological parameters to predict EC's T‐stage diagnosis, which help for thoracic surgeons and radiologists to T‐stage diagnose EC with CTA, and to make prognosis and treatment decisions.^37^

## 2 MATERIALS AND METHODS

### 2.1 General information

**FIGURE 1:**
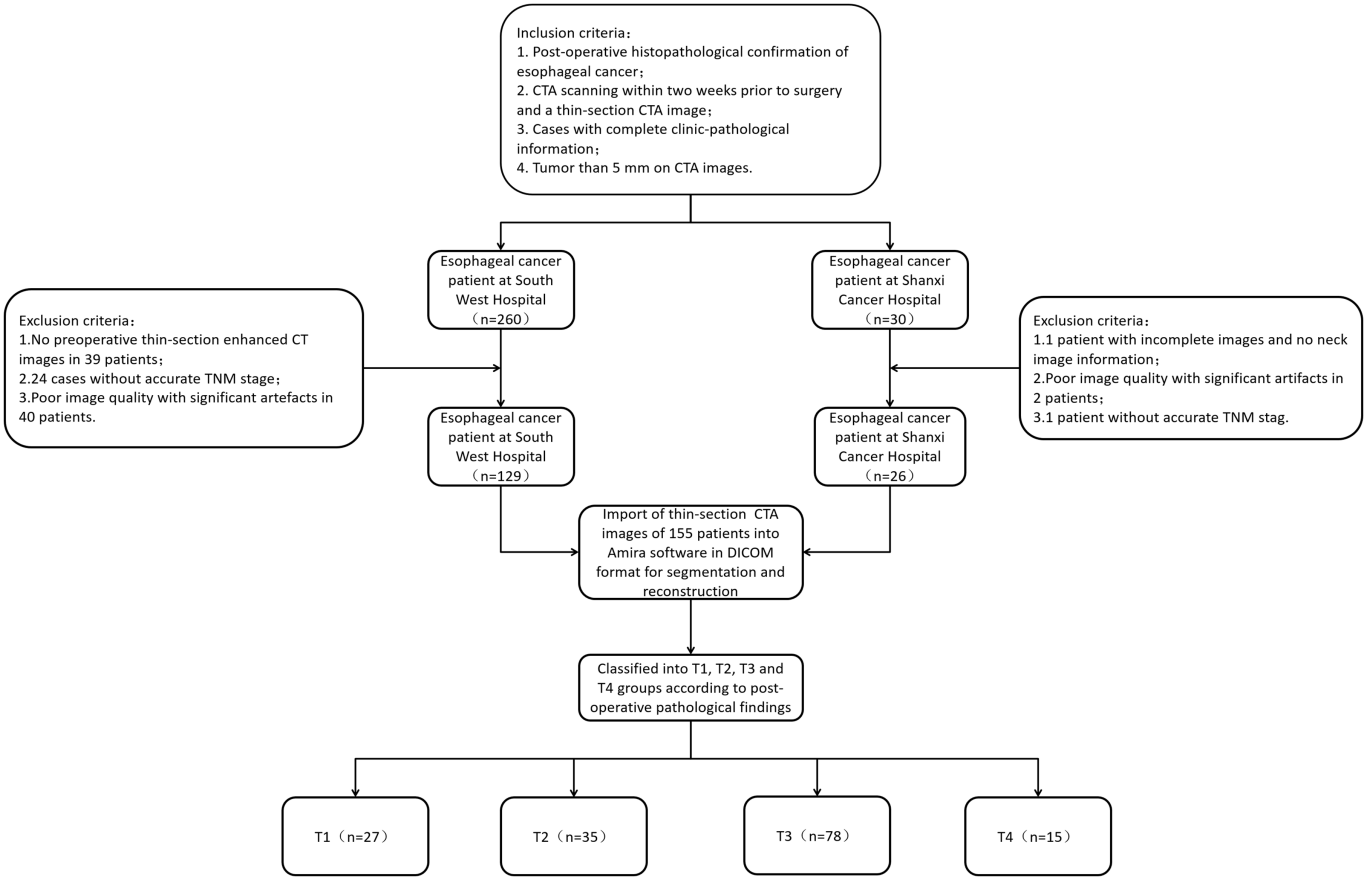
Flowchart of patient inclusion or exclusion.

This study retrospectively collected data from patients who underwent radical EC surgery at the First Affiliated Hospital of the Army Medical University and Shanxi Cancer Hospital between January 2018 and April 2022 and had post‐operative histopathological confirmation of EC. Inclusion criteria^18^: (1) Post‐operative histopathological confirmation of EC; (2) CTA scan within 2 weeks before surgery and thin CTA images; (3) cases with complete clinical‐pathological information; (5) tumour lesions >5 mm on CTA images. Exclusion criteria were as follows^19^: (1) no accurate clinical‐pathological information, or some missing information; (2) no pre‐operative CTA image scan in hospital; (3) poor image quality or artefacts that significantly affect segmentation; (4) no thin CTA images or incomplete image information (e.g. images lack data on the neck or gastro‐oesophageal junction, etc.); (5) no obvious lesions on CTA images (lesions <5 mm, etc.) (Figure 1).^20^, ^21^

According to the above inclusion and exclusion criteria, 155 patients with EC were included in this study and divided into four groups according to their T‐stage. Differences in the general condition of the four groups were not statistically significant (*p* > 0.05) and were comparable (Table 1).^21^, ^22^

**TABLE 1:** Information regarding the characteristics of patients with different T‐stages of EC.

Characteristics	T1 (*n* + %)	T2 (*n* + %)	T3 (*n* + %)	T4 (*n* + %)	*p*
Age (years)	60.15 ± 5.50	61.23 ± 7.56	61.90 ± 8.54	65.00 ± 6.88	0.27
Gender					0.43
Male	24 (88.9)	29 (82.9)	69 (88.5)	11 (73.3)	
Female	3 (11.1)	6 (17.1)	9 (11.5)	4 (26.7)	
Tumour location					0.47
Upper	9 (33.3)	10 (28.6)	17 (21.8)	2 (13.3)	
Middle	16 (59.3)	20 (57.1)	47 (60.3)	12 (80.0)	
Lower	2 (7.4)	5 (14.2)	14 (17.9)	1 (6.7)	
N stage					0.13
0	24 (88.9)	24 (68.6)	42 (53.8)	12 (80.0)	
1	3 (11.1)	7 (20.0)	23 (29.5)	1 (6.7)	
2	0	2 (5.7)	10 (12.8)	1 (6.7)	
3	0	2 (5.7)	3 (3.8)	1 (6.7)	
Degree of pathological differentiation					0.10
I	5 (18.5)	3 (8.6)	14 (17.9)	2 (13.3)	
II	21 (77.8)	21 (60.0)	47 (60.3)	9 (60.0)	
III	1 (3.7)	11 (31.4)	17 (21.8)	4 (26.7)	

*Note*: Data are expressed as number (%) and mean ± SD, depending on the variable distribution.

Abbreviation: EC, oesophageal cancer.

### 2.2 CTA image acquisition

All patients who participated in this study underwent appropriate breathing exercises, and a 64‐bit multi‐detector spiral CTA scanner was used to scan all images. All patients were placed in the supine position with the head facing forward and the hands above the head, and either the chest or the upper abdomen was scanned during the CTA examination.^22^ Typically, a traditional CTA scan is performed first, followed by intravenous injection of contrast medium using a syringe pump.^23^ Thin‐layer CTA images were exported in the DICOM format from the workstation when the scan was finished.

### 2.3 Image pre‐processing and segmentation

To obtain a 3D reconstructed model of the EC for further analytical studies, thin‐CTA images of 1–2 mm thickness exported from the workstation in DICOM format were uploaded to Amira software (v.6.0.0) for manual segmentation of the EC images. The SEGMENTATION module of the software was used to segment EC images. The segmented structures included the tumour, normal oesophagus, pericardium, aorta, bronchi and lung tissue, creating labels for a total of seven structures. For the outlining of each tumour region, EC contours were drawn around the total tumour volume, and any pixels <−50 HU were excluded to avoid interference with the outlining of the tumour boundary from the surrounding adjacent air, fat, blood vessels (including foci of calcification in the canal wall), and bone. When there was uncertainty concerning the tumour area, imaging physician's judgement prevailed.

### 2.4 3D EC reconstruction model

Once image segmentation is complete, the original and segmented images were saved separately in “.am” format. 3D models were reconstructed using the Generate Surface in Amira software, which allows a 3D model of EC to be viewed in all directions, moved, scaled and rotated. Three‐dimensional reconstruction and visualisation can directly display the tumour's location, 3D shape and spatial relationship. Based on the 3D model, we can calculate morphological parameters of the tumour, which can help doctors identify different T stages of tumour more accurately.

### 2.5 Data measurement based on 2D and 3D reconstruction models

In the 3D EC reconstruction models, the surface area volume module in Amira software was used to measure the surface area and volume of the tumour and surrounding normal tissue and to analyse the volume relationships between tumour and total oesophageal at different T‐stages. The tumour‐related lengths were measured on the 3D model using Measure: line. The measurements included the EC surface area, volume, major axis (defined as the longest distance of the tumour in coronal position measured on the basis of 3D reconstruction of the tumour.), minor axis (defined as the longest distance of the tumour in sagittal position measured on the basis of 3D reconstruction of the tumour.), longitudinal length, normal oesophageal surface area, volume and longitudinal length. To exclude the influence of chance on the results, four sections were randomly selected for measurement of the major and minor axis of the oesophageal tumour and normal oesophagus. Tumour roughness and contact with the aorta were assessed based on the reconstructed 3D model and coronal, sagittal and axial CTA images.

### 2.6 Statistical analysis

#### 2.6.1 Correlation study of morphological variations between different EC T‐stages

The above measurement and assessment results were analysed using SPSS software (https://www.ibm.com/cn‐zh/spss) for relevant statistics. Basic information surrounding cases was expressed using means ± standard deviation, one‐way ANOVA was used for comparison between multiple groups, and for independent samples *t*‐test was used for analysis between two groups; differences were considered statistically significant at *p* < 0.05.^24^, ^25^, ^26^

#### 2.6.2 Calculation of cut‐off values

The ROC is a curve plotted with a false positive rate (1‐specificity) as the horizontal coordinate (X‐axis) and the true positive rate (sensitivity) as the vertical coordinate (Y‐axis).^27^, ^28^ The AUC, ROC, sensitivity (S) and specificity (P) of each measurement were analysed, with an AUC >0.6 being considered clinically significant. The Jorden index, sensitivity + specificity, also known as the cut‐off value, is a commonly used measure of the diagnostic validity of a disease.^29^ It is calculated by plotting the ROC and assigning equal weight to sensitivity and specificity to identify the optimal cut‐off point, thus maximising sensitivity and specificity.^29^, ^30^

#### 2.6.3 Measure based on 3D‐model T staging evaluation

We invited two radiologists (the first with 5 years and the second with 10 years of experience) to independently re‐evaluate the data and their results were compared with the measure based on 3D‐model predictions, using ROC curves and AUCs to evaluate.

## 3 RESULTS

### 3.1 Patient characteristics and the 3D reconstruction of EC

**FIGURE 2:**
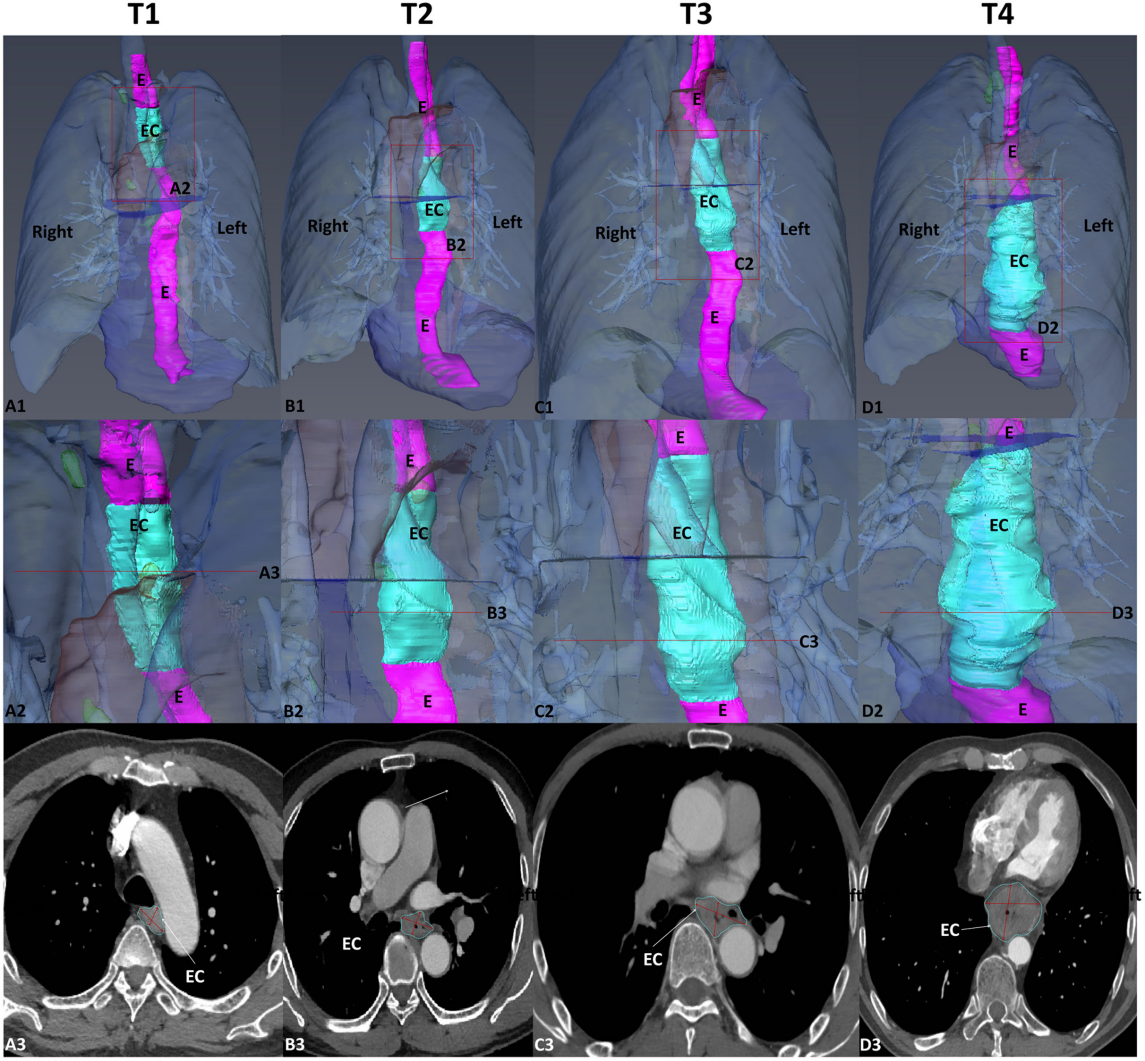
3D reconstructed model of EC. A1–D1:3D reconstruction of the four stages; A2–D2: local enlargements of the models; A3–D3: Transverse CTA images of the EC, including lines measuring the major and minor axis. E, normal oesophagus, EC, oesophageal cancer.

A total of 155 patients with EC were enrolled in this study, and their characteristics are detailed in Tables 1 and 2. According to the AJCC 8th edition staging criteria, T1 28 (18.1%) were T2 35 (22.6%) were T3 78 (50.3%) were T4 14 (9.0%). To better present the 3D EC reconstruction model, we randomly selected one case from each T‐stage for demonstration (Figure 2).

**TABLE 2:** Statistical results of correlations between different T‐stages of EC.

Characteristics	T1 (27)	T2 (35)	T3 (78)	T4 (15)	Count	Percentage	*F*	*p*
Depth of tumour infiltration							123.70	0.001
Invasion of mucosal muscle layer	8 (29.6)	1 (2.9)	0	0	9	5.8		
Invasion of submucosal layer	18 (66.7)	5 (14.3)	2 (2.6)	1 (6.7)	26	16.8		
Invasion of superficial muscular layer	1 (3.7)	13 (37.1)	3 (3.8)	0	17	11.0		
Invasion of deep muscular layer	0	16 (45.7)	16 (20.6)	1 (6.7)	33	21.3		
Invasion of outer membrane layer	0	0	54 (69.2)	5 (33.3)	59	38.1		
Penetration of the outer membrane	0	0	3 (3.8)	8 (53.3)	11	7.1		
Neurovascular invasion							8.78	0.001
No invasion seen	27 (100)	27 (77.1)	57 (73.1)	5 (33.3)	116	74.8		
Presence of neurovascular invasion	0	8 (22.9)	21 (26.9)	10 (66.7)	39	25.2		
Type of pathology							3.43	0.020
Constricted	2 (7.4)	0	0	0	2	1.3		
Myxomatous	2 (7.4)	2 (5.7)	0	2 (13.3)	6	3.9		
Ulcerated	14 (51.9)	24 (68.6)	46 (59.0)	11 (73.3)	95	61.3		
Medullary	9 (33.3)	9 (25.7)	32 (41.0)	2 (13.3)	52	33.5		
Roughness							3.42	0.020
1: Smooth	3 (21.4)	2 (7.7)	10 (19.2)	0	15	15		
2: Slightly coarse	5 (35.7)	14 (53.8)	21 (40.4)	2 (25.0)	42	42		
3: Severely coarse	6 (42.9)	10 (38.5)	21 (40.4)	6 (75.0)	43	43		
Relationship with the aorta							5.267	0.002
0: No contact	0	1 (3.8)	0	0	1	1		
1: Contact, but not squeeze	10 (71.4)	11 (42.3)	21 (40.4)	0	42	42		
2: Squeeze slightly	2 (14.3)	12 (46.2)	20 (38.5)	3 (37.5)	37	37		
3: Squeeze severely	2 (14.3)	2 (7.7)	11 (21.2)	5 (62.5)	20	20		
Clinical stage							61.57	0.001
I	24 (88.9)	0	0	0	24	15.5		
II	3 (11.1)	24 (68.6)	43 (55.1)	0	70	45.2		
III	0	9 (25.7)	32 (41.0)	13 (86.7)	54	34.8		
IV	0	2 (5.7)	3 (3.9)	2 (13.3)	7	4.5		

*Note*: Data are expressed as number (%), depending on the variable distribution.

Abbreviation: EC, oesophageal cancer.

We found that there was a significant positive correlation between the depth of tumour infiltration and the T‐stage of EC (*p* < 0.05), with a strong correspondence. The proportion of neurovascular invasion gradually increased with elevating T‐stage of EC, with 22.9% in T2, 26.9% in T3 and 66.7% in T4, where it can be seen that the proportion is gradually increasing. The ulcerative type accounted for the majority (61.3%), followed by the medullary type (33.5%), mainly in the T3 and T4 stages of EC; the pathological types of advanced EC were mainly ulcerative and medullary; the roughness of EC increased with the T stage of EC, and the roughness of T4 stage EC, with a score of 3 accounting for 75%. The relationship between tumour and aorta can be used to evaluate the risk level of surgery; we found that the score of the relationship between tumour and aorta in T1 was mainly 1 (71.4%), indicating a low‐risk level at surgery; the score of the relationship between tumour and aorta in T2 and T3 was 1–2, with a higher risk level at surgery than in T1; and the score of T4 was mainly 3 (62.5%), indicating greater risk. Clinical staging was judged according to TNM stage, the clinical stage of T1 was mainly (88.9%) andII(11.1%), the clinical stage of T2 and T3 was mainly concentrated in II, III and IV, the proportion of II was 68.6% and 58.1%, the proportion of III and IV in T4 was 86.7% and 13.3%. Hence, with the increase of T staging, the clinical stage also increased with it, and the severity was higher (Table 1 and 2).

### 3.2 Measurement and comparison of different T‐stage 3D models of EC

**FIGURE 3:**
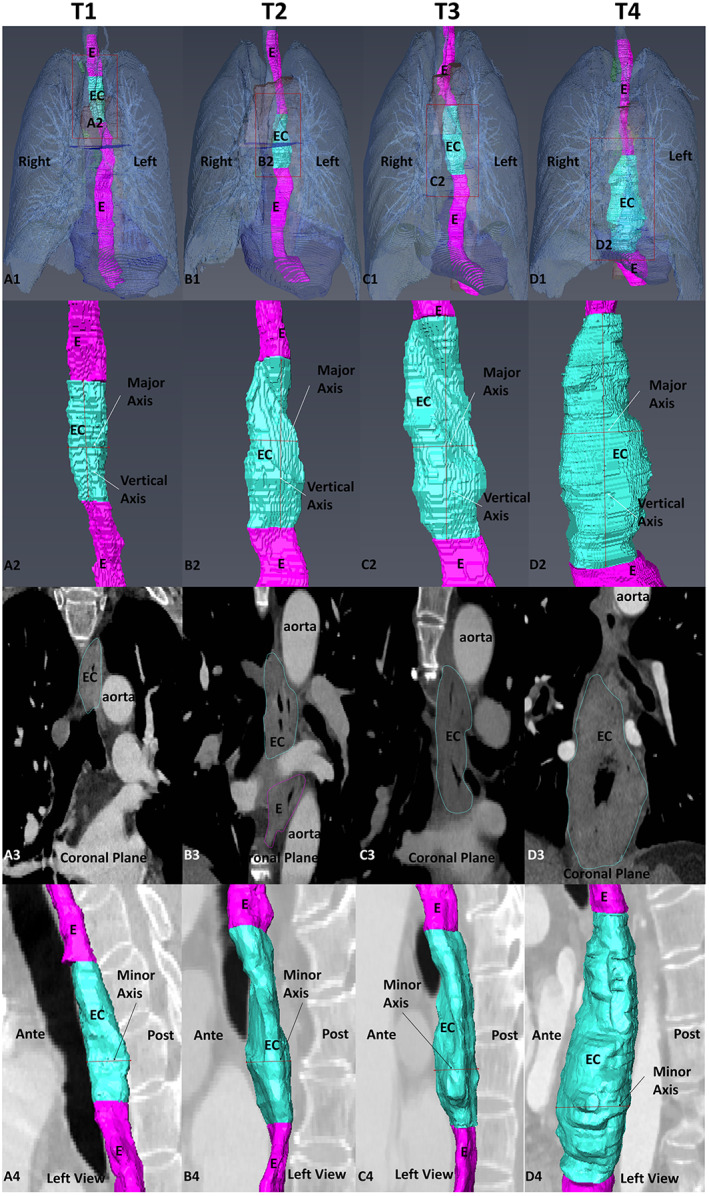
Measurement of tumour roughness and major and minor axis at different T‐stages of EC. A1–D1: 3D reconstructed models; A2–D2: local magnifications, which can determine the roughness and measure the length of the major axis and vertical axis of the EC; A3–D3: coronal CTA images of the tumour area, which can easily show the roughness of the tumour and its position relative to surrounding tissues; A4–D4: sagittal images of the reconstructed tumour superimposed on normal oesophagus (left‐side view), mainly used to measure the minor axis of the tumour. E, normal oesophagus, EC, oesophageal cancer.

The mean major and minor axis of oesophageal tumours were 22.06 ± 2.0, 26.06 ± 5.10, 30.21 ± 6.70, 33.99 ± 9.49, and 13.97 ± 2.7, 16.89 ± 3.44, 20.61 ± 5.47, and 26.69 ± 7.92 mm for the T‐4 stages, respectively, and the differences in values were statistically significant (*p* < 0.05) (Figure 3). The surface areas of T1–T4 were with an increasing trend, where statistically significant for T1:T2, and T3:T4 (*p* = 0.044, *p* = 0.022). Tumour volumes for T1–T4 were 12,934.36 ± 7739.25, 23,095.27 ± 14,975.67, 37,577.98 ± 36,085.64, and 58,579.25 ± 41,073.96 mm^3^, showing an increasing relationship, in which the EC volumes of patients with EC in T1:T2, T2:T3 were statistically different (*p* = 0.029, *p* = 0.047); the EC longitudinal length, roughness score and EC volume/total oesophageal volume of patients with EC; there were no statistically significant differences between the above three parameters in the T1–T4 (*p* > 0.05). Tumour surface area/total surface areathe of oesophagus, tumour major‐axis length/total lengththe of oesophagus, and EC relationship with the aorta in EC patients at T3–T4 were all *p* < 0.05 (Table 3).

**TABLE 3:** Measurement data related to different T‐stages of EC and comparative results.

	T1	T2	*p*	T2	T3	*p*	T3	T4	*p*
EC Longitudinal axis length (mm)	50.70 ± 17.24	61.13 ± 23.67	0.155	61.13 ± 23.67	63.58 ± 21.88	0.651	63.58 ± 21.88	78.95 ± 25.57	0.075
EC mean major axis (mm)	22.06 ± 2.09	26.06 ± 5.10	**0.008**	26.06 ± 5.10	30.21 ± 6.70	**0.010**	30.21 ± 6.70	33.99 ± 9.49	**0.037**
EC mean minor axis (mm)	13.97 ± 2.73	16.89 ± 3.44	**0.009**	16.89 ± 3.44	20.61 ± 5.47	**0.003**	20.61 ± 5.47	26.69 ± 7.92	**0.009**
EC surface area (mm^2^)	4493.07 ± 1998.15	6462.54 ± 3206.27	**0.044**	6462.54 ± 3206.27	7727.38 ± 3900.41	0.157	7727.38 ± 3900.41	11,488.13 ± 5944.26	**0.022**
EC volume (mm^3^)	12,934.36 ± 7739.25	23,095.27 ± 14,975.67	**0.029**	23,095.27 ± 14,975.67	37,577.98 ± 36,085.64	**0.047**	37,577.98 ± 36,085.64	58,579.25 ± 41,073.96	0.138
EC Roughness	2.21 ± 0.80	2.31 ± 0.62	0.684	2.31 ± 0.62	2.21 ± 0.75	0.574	2.21 ± 0.75	2.75 ± 0.46	0.054
EC Relationship with the aorta	1.43 ± 0.76	1.58 ± 0.70	0.539	1.58 ± 0.70	1.81 ± 0.77	0.202	1.81 ± 0.77	2.63 ± 0.52	**0.005**
EC surface area/total oesophageal surface area	0.25 ± 0.09	0.31 ± 0.11	0.099	0.31 ± 0.11	0.33 ± 0.12	0.355	0.33 ± 0.12	0.44 ± 0.16	**0.029**
EC volume/total oesophageal volume	0.26 ± 0.11	0.34 ± 0.14	0.065	0.34 ± 0.14	0.40 ± 0.17	0.122	0.40 ± 0.17	0.52 ± 0.21	0.074
EC longitudinal axis length/total oesophageal length	0.22 ± 0.08	0.25 ± 0.09	0.300	0.25 ± 0.09	0.25 ± 0.08	0.905	0.25 ± 0.08	0.33 ± 0.12	**0.019**

The significance of bold values was more intuitive display of data with *p* less than 0.05.

*Note*: Data are expressed as mean ± SD, depending on variable distribution. The relationship between tumour and aorta score: (1) 0 point: the tumour and the aorta had no contact at all or were separated completely; (2) 1 point: the tumour and the aorta were in contact with each other, and there is no tumour extrusion to the aorta; (3) 2 points: the tumour and the aorta were in contact with each other, and there is one slight tumour extrusion to the aorta; (4) 3 points: the tumour extrudes the aorta seriously, or there is two or more tumour extrusion to the aorta.

Abbreviation: EC, oesophageal cancer.

The roughness scores for oesophageal tumours were based on a CTA 3D reconstruction model of EC combined with CTA images in the transverse, coronal and sagittal planes (Figure 3). We found that the roughness of the T1–T4 stages was 2.21 ± 0.80, 2.31 ± 0.62, 2.21 ± 0.75 and 2.75 ± 0.46, respectively, with an increasing trend.

**FIGURE 4:**
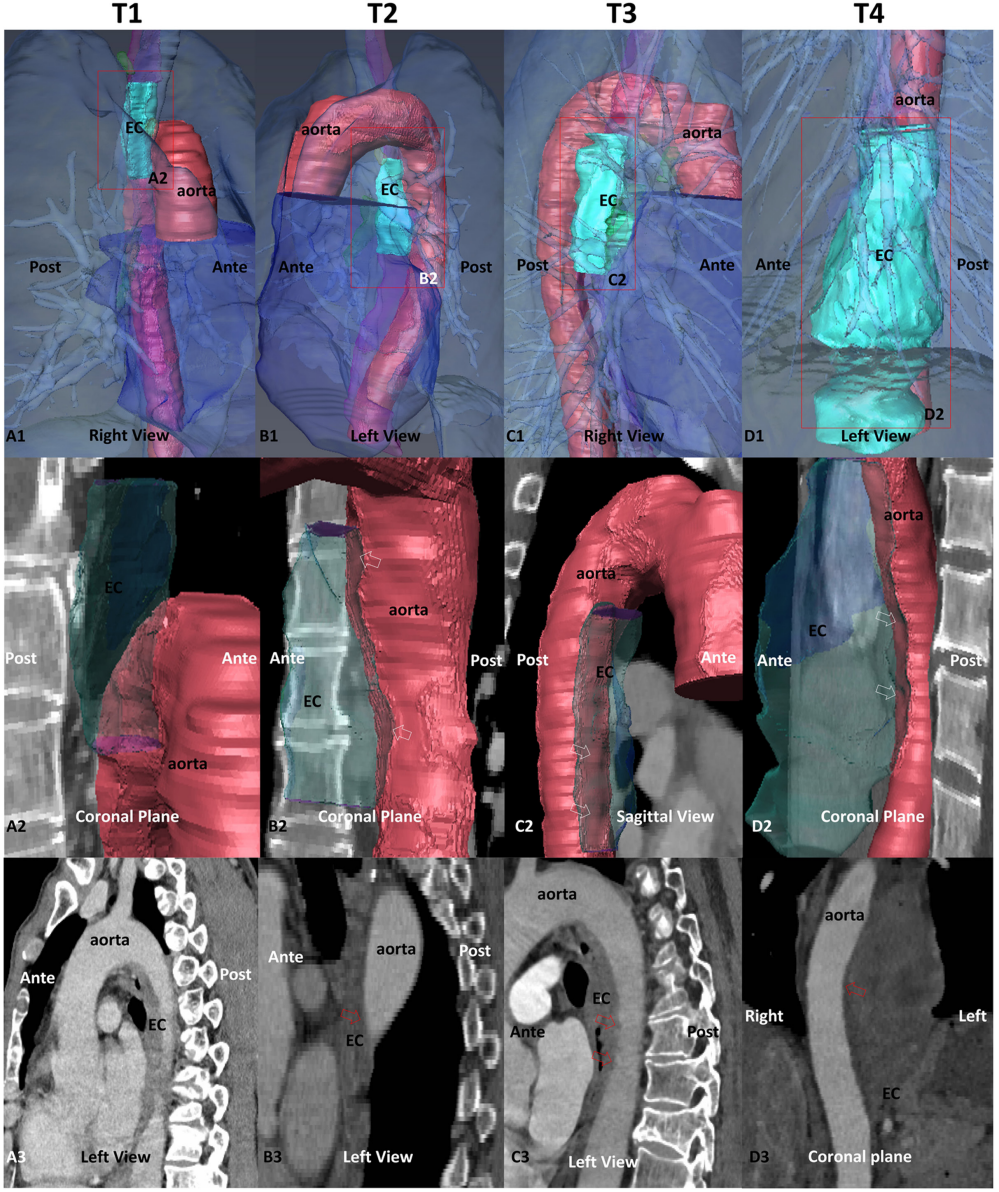
Relationship between tumour and aorta in the 3D reconstructed model. A1–D1 are 3D reconstructions of EC and its adjacent structures; A2–D2 are 3D reconstructed tumours (with transparency treatment) with superimposed sagittal CTA images of the aorta, allowing easier assessment of the relative position of the tumour in relation to the aorta, and also affording clear visualisation of aortic vessel wall compression by the tumour; A3–D3 are sagittal and coronal CTA images of the tumour region. E, normal oesophagus, EC, oesophageal cancer.

The relationship between the tumour and aorta was scored using a four‐point scale, in which the relative positions of the tumour and aorta were observed on the 3D model and scored in conjunction with CTA images (Figure 4). We found that the results for EC in relation to the aorta at T1–T4 were 1.43 ± 0.76, 1.58 ± 0.70, 1.81 ± 0.77 and 2.63 ± 0.52, exhibiting an increasing trend, with a statistical difference of *p* < 0.05 at T3–T4.

### 3.3 ROC curve analysis and calculation of cut‐off values

**FIGURE 5:**
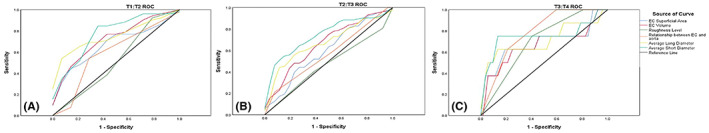
ROC curves for different T‐stage related indicators of oesophageal cancer. A is the ROC curve for T1:T2, B is the ROC curve for T2:T3, and C is the ROC curve for T3:T4.

By ROC analysis, there were no significant differences in EC long axis length, roughness score and relationship with aorta between different T‐stages of EC, while there were significant differences in EC surface area, EC volume, mean major axis and mean minor axis between different T‐stages of EC (Figure 5, Tables 4).

**TABLE 4:** AUC, sensitivity, specificity and cut‐off values for different stages of EC.

Measurement indicators	AUC	S	P	Cut‐off value
T1:T2				
EC surface area	0.673	0.769	0.571	4008.50
EC volume	0.728	0.769	0.643	11,712.00
Roughness	0.522	0.385	0.571	2.50
Relationship with aorta	0.585	0.538	0.714	1.50
Mean major axis	0.761	0.615	0.857	24.25375
Mean minor axis	0.775	0.846	0.714	14.69
T2:T3				
EC surface area	0.601	0.635	0.538	5533.50
EC volume	0.663	0.654	0.615	19,809.00
Roughness	0.473	0.404	0.615	2.50
Relationship with aorta	0.571	0.212	0.923	2.50
Mean major axis	0.675	0.654	0.615	26.2825
Mean minor axis	0.744	0.808	0.538	16.90625
T3:T4				
EC surface area	0.683	0.625	0.827	10,894.50
EC volume	0.663	0.625	0.769	44,103.50
Roughness	0.697	0.750	0.596	2.50
Relationship with aorta	0.782	0.625	0.788	2.50
Mean major axis	0.709	0.625	0.885	37.0475
Mean minor axis	0.748	0.750	0.885	25.46375

Abbreviations: AUC, area under the curve; EC, oesophageal cancer; P, specificity; S, sensitivity.

The T1:T2's AUC for EC surface area, EC volume, major axis and minor axis were 0.673, 0.728, 0.761 and 0.775, respectively. All values were >0.6, indicating that they correlated more with the T‐stage of EC, but the AUC for roughness and relationship with the aorta were 0.522 and 0.585, respectively, with values <0.6, which correlated poorly (Figure 5A).

Next, T2 and T3 were analysed, and by comparing AUC, S and P, we found that the EC surface area, volume, mean major and minor axis were clinically significant in these two stages and their AUCs were all >0.6; however, the roughness and the relationship with aortic area was <0.6 (0.473, 0.571) and the correlation was poor (Figure 5B).

Finally, by analysing the T3 and T4 periods, we found high correlations with AUCs >0.6 for all parameters (0.683, 0.663, 0.697, 0.782, 0.709, 0.748) (Figure 5C).

Table 5 summarises the predictive results of the T‐stage of EC in combination with the critical values. The EC T1:T2, T2:T3, T3:T4 volume cut‐off values were 11,712.00, 19,809.00, 44,103.50 mm^3^, with mean major axis and mean minor axis cut‐off values of 24.25, 26.28, 37.05 and 14.69, 16.91, 25.46 mm, and EC surface areas of 4008.50, 5533.50 and 10,894.50 mm^2^.

**TABLE 5:** Cut‐off values of morphological parameters for different T‐stages of EC.

	T1:T2	T2:T3	T3:T4
EC volume (mm^3^)	11,712.00	19,809.00	44,103.50
Mean major axis (mm)	24.25	26.28	37.05
Mean minor axis (mm)	14.69	16.91	25.46
EC surface area (mm^2^)	4008.50	5533.50	10,894.50

Abbreviation: EC, oesophageal cancer.

### 3.4 Measure based on 3D‐model T staging evaluation

**FIGURE 6:**
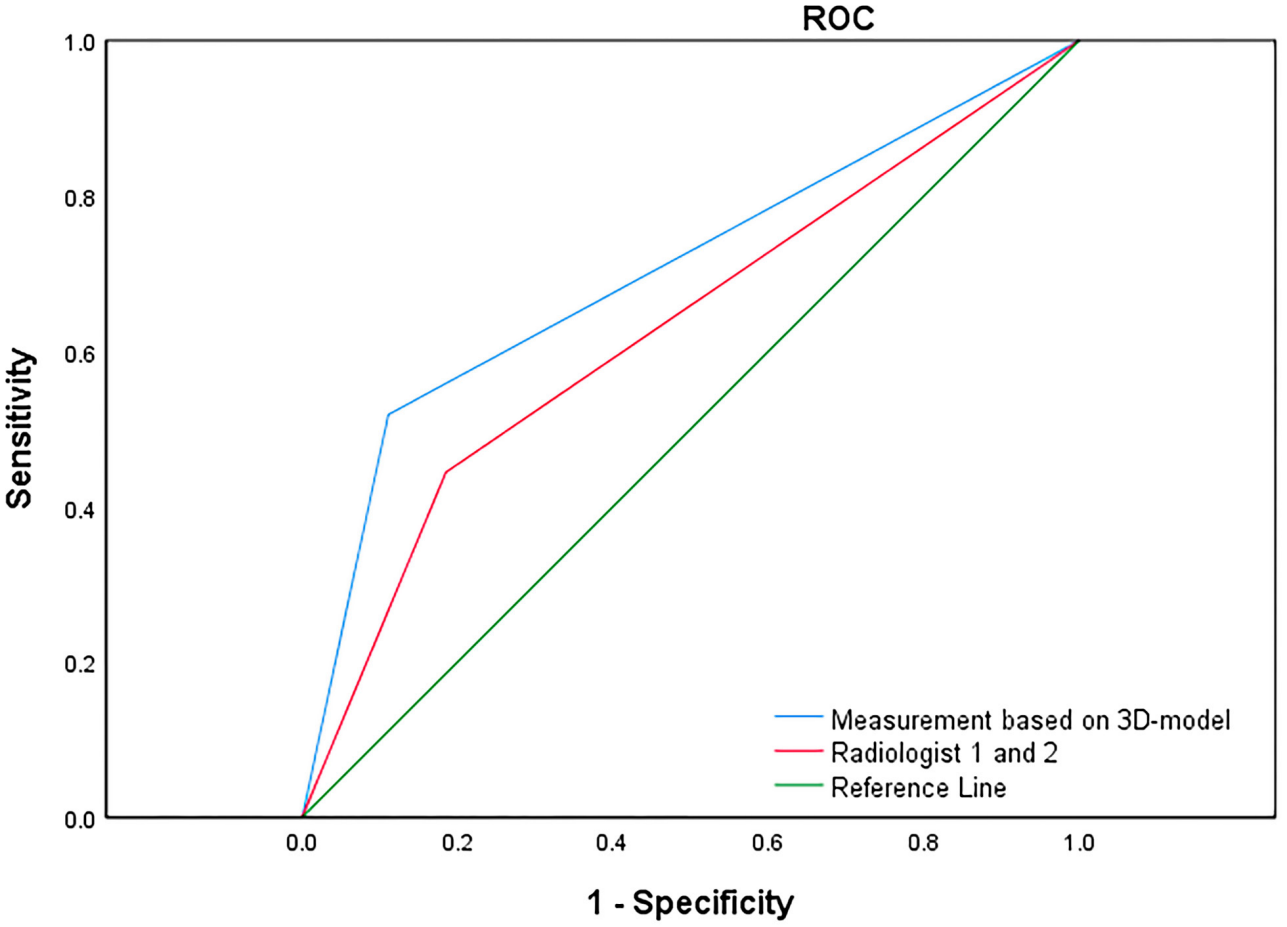
ROC curves and AUC of two radiologists and measurement based on 3D‐model.

We found that the model results were better overall than the radiologists' diagnoses. By comparison, radiologists predicted different T‐stages of oesophageal cancer with an AUC value of 0.630 and the accuracy of 0.760, 0.443, 0.352 and 0.167 for T1–T4 stage of EC. Our 3D model‐based measurements had an AUC value of 0.704, higher than radiologists and the accuracy of 0.673, 0.782, 0.601, 0.752 for T1–T4 stage of EC. (Table 6) The ROC curves of the two physicians are shown in Figure 6.

**TABLE 6:** Two radiologists and measurement based on 3D‐model T staging of precision and AUC.

T stage	Precision of radiologists' diagnosis	Precision of measurement based on 3D‐model	AUC of radiologists' diagnosis from T1–T4	AUC of measurement based on 3D‐model from T1–T4
T1	0.760	0.673	0.630	0.704
T2	0.443	0.782		
T3	0.352	0.601		
T4	0.167	0.752		

## 4 DISCUSSION

In this study, based on 3D reconstruction technique, the imaging morphological features and TNM staging criteria, we retrospectively used CTA images of 155 patients with EC to construct 3D models, as well as to measure the relevant indices of EC and to conduct statistical analysis to determine the correlation between them and T‐stage. In summary, EC volume, EC surface area, mean major axis and mean minor axis were clinically significant in predicting the T‐stage of EC, and roughness and relationship to the aorta had poor predictive results in early T‐stage EC.

Our study showed that EC surface area, EC volume, mean major axis and mean minor axis were all significantly different between different EC T‐stages (*p* < 0.05), which agrees with Zhao et al.'s study.^31^ However, their measurements only included tumour volume, length and CTA value and did not include the mean major axis, mean minor axis, roughness or relationship with the aorta of the tumour. Therefore, our study uses more measurements to predict EC's T‐staging diagnosis more comprehensively and accurately. Especially in T4 stage, the tumour has clearly broken through the plasma membrane layer of the oesophagus, invading adjacent structures around the oesophagus. The tumour develops rapidly after breaking through the plasma membrane layer.

In our study, we found that the mean major and minor axis of the tumour (*p* < 0.05) had greater value in predicting the T‐stage of EC; however, the length of the tumour in the longitudinal axis (*p* > 0.05) was not significantly different between the different stages, consistent with the findings of Zhang et al.,^32^ who concluded by correlation analysis that there was no significant correlation between oesophageal tumour's length and prognosis. Xu et al.^37^ used the barium esophagography and computed tomography to determine the oesophageal tumour length and diameter before treatment. They found that tumour length and diameter are both independent prognostic factors for ESCC patients treated with definitive (chemo)radiotherapy, consistently with our results. We speculate that this may be related to the invasive growth of EC, which infiltrates mainly in the horizontal direction and less so along the longitudinal axis, making the mean long and short tumour diameters a reliable basis for assessing tumour T‐staging. Among EC volume, major axis and minor axis, we found that the minor axis is the preferred parameter in evaluating tumour depth. According to the results of multifactorial analysis, the EC volume, major axis and minor axis have *p* < 0.05 in distinguishing different T stages, and the AUC values of the minor axis are the highest. In comparison, the minor axis has more predictive value. Simopoulos et al.^33^ also used this method to help clinicians to diagnose prostate cancer by using MRI images to measure nucleus length and tumour volume in targeted biopsies and by analysing indicators that correlated with pathological staging. They concluded that when the nucleus length exceeded 10 mm, prostate cancer is easy to spread. Chen et al.^34^ used CTA images and developed and validated an integrated gross tumour volume (GTV)‐TNM stratification system for unrespectable locally advanced non‐small cell lung cancer (LANSCLC) based on the possibility that the GTV is an independent prognostic factor for unrespectable LANSCLC treated with concurrent radiotherapy. Therefore, the volume and the mean major and minor axis of EC are significantly predictable to the T‐stage of the tumour.

Next, we quantified the T‐stage of EC based on the Jorden index and other relevant calculations. The more advanced the stage, the greater the critical value, indicating that the higher the T‐stage of the tumour, the greater the increase in volume. Chen et al.^35^ used cut‐off values to help physicians diagnose the EC's radiotherapy efficacy. They analysed patients with non‐metastatic and unrespectable ESCC who underwent pre‐operative FDG PET/CT and were treated with chemoradiotherapy, and used ROC analysis to determine the optimal cut‐off value for the primary tumour (NTR). A cut‐off value of 0.46 was obtained for NTR with an AUC of 0.648. Gao et al.^36^ used endoscopic ultrasound (EUS) images to calculate the tumour size threshold for gastrointestinal stromal tumours (GIST) by ROC for use in pre‐operative treatment strategies and appropriate timing of follow‐up for GIST. They finally obtained an AUC of 0.818 for patients with 1, 2 or more years of follow‐up and an optimal cut‐off value of 9.5 mm for tumour size. With this cut‐off value, we can determine the T‐stage of EC by calculating the tumour's volume, cross‐sectional major and minor axis. Pre‐operative T‐stage diagnosis is very important to thoracic surgeons because it is used to determine whether to have surgery directly or have neoadjuvant chemotherapy before surgery. Our study is able to overcome, to a certain extent, the difficulty for thoracic surgeons in T‐stage diagnosing EC by CTA, which helps to accurate T‐stage diagnosis and guides prognostic assessment and treatment decisions.

Finally, we found that the accuracy of T1 stage radiologists was higher than the measurement model, and T2–T4 stage was lower than the model. We speculate that the accuracy of T2–T4 stage is poorer because radiologists have no relative concept of tumour size at different T stages and only classify by experience. About T1 stage, measurement based on 3D‐model has poor precision, because the size of EC in T1 is very small and the segmentation error exists. In contrast, we used the volume, surface area, long diameter and short diameter of EC to T‐stage diagnose EC more objectively and more accurately. Therefore, when compared with two experienced radiologists, the precision of measurement based on 3D‐model was better than theirs, indicating that the model is of good clinical use and can help radiologists to improve their efficiency.

However, our study also had certain limitations. First, although we selected data from two hospitals for multi‐centre study, the relatively small quantity of data from Shanxi Cancer Hospital led to uneven data, which may have influenced the results. Second, the number of patients included in the study was relatively small, and we will continue to expand the sample size for in‐depth validation analysis in the future. Third, the scores of roughness and relationship between tumour and aorta are less objective, after which we will seek more objective factors to replace them, such as CT values.

## 5 CONCLUSION

In summary, our study based on CTA 3D reconstruction of EC is helpful for accurately determining the tumour location in EC, clearly and comprehensively observing the location, 3D shape and the relationship between the tumour and its adjacent structures. Volume and the major and minor axis of EC are significantly predictable factors to the T‐stage diagnosis of the tumour, and we could calculate the volume, the major and minor axis of EC based on CTA to T‐stage diagnose EC before surgery.

## AUTHOR CONTRIBUTIONS

**Runyuan Wang:** Data curation (lead); formal analysis (lead); methodology (equal); software (lead); visualization (lead); writing – original draft (lead). **Xiaoqin Zhang:** Methodology (equal); supervision (equal); writing – review and editing (equal). **Wei Wu:** Funding acquisition (equal); methodology (equal); project administration (equal). **Jinfeng Ma:** Data curation (equal); resources (equal). **Jincheng Chen:** Data curation (equal); resources (equal). **Zhu Zhang:** Data curation (equal). **Liqun Liu:** Data curation (supporting); formal analysis (supporting). **Shanshan Xu:** Formal analysis (supporting). **Ximei Cao:** Writing – review and editing (supporting). **Yi Wu:** Conceptualization (equal); funding acquisition (equal); methodology (equal); supervision (equal); writing – review and editing (lead). **Huilin Cui:** Funding acquisition (equal); writing – review and editing (equal).

## FUNDING INFORMATION

This study has received funding by the National Natural Science Foundation of China (31971113), the Chongqing Science and Technology Talent Project (CQYC201905037), University Funded Science and Technology Innovation Capacity Improvement Project (2019XYY14), Chongqing Scientific and Health Committee Joint Research Key Project (2022ZDXM018), Basic Research (Free Exploration) Project of Shanxi Province (2021011079‐2) and Chongqing Excellence Package Project (Project Approval Number: cstc2022ycjh‐bgzxm0071).

## CONFLICT OF INTEREST STATEMENT

The authors have no conflict of interest.

## ETHICS STATEMENT

The study protocol was approved by the Ethics Committee of the First Affiliated Hospital of Army Military Medical University (No. (B)KY2021165). Informed consent was objectively unavailable for this study, and subjects' privacy and personally identifiable information were protected.

## Data Availability

The data that support the findings of this study are available from the corresponding author, [Runyuan Wang], upon reasonable request.
